# Explainable artificial intelligence in deep learning–based detection of aortic elongation on chest X-ray images

**DOI:** 10.1093/ehjdh/ztae045

**Published:** 2024-06-25

**Authors:** Estela Ribeiro, Diego A C Cardenas, Felipe M Dias, Jose E Krieger, Marco A Gutierrez

**Affiliations:** Heart Institute (InCor), Clinics Hospital University of Sao Paulo Medical School (HCFMUSP), Av. Dr. Enéas Carvalho de Aguiar, 44 - Cerqueira César, São Paulo, SP 05403-900, Brazil; University of Sao Paulo Medical School (FMUSP), Av. Dr. Arnaldo, 455 - Cerqueira César, Pacaembu, SP 01246-903, Brazil; Heart Institute (InCor), Clinics Hospital University of Sao Paulo Medical School (HCFMUSP), Av. Dr. Enéas Carvalho de Aguiar, 44 - Cerqueira César, São Paulo, SP 05403-900, Brazil; Heart Institute (InCor), Clinics Hospital University of Sao Paulo Medical School (HCFMUSP), Av. Dr. Enéas Carvalho de Aguiar, 44 - Cerqueira César, São Paulo, SP 05403-900, Brazil; Polytechnique School, University of Sao Paulo (POLI USP), Av. Prof. Luciano Gualberto, 380 - Butantã, São Paulo, SP 05508-010, Brazil; Heart Institute (InCor), Clinics Hospital University of Sao Paulo Medical School (HCFMUSP), Av. Dr. Enéas Carvalho de Aguiar, 44 - Cerqueira César, São Paulo, SP 05403-900, Brazil; Heart Institute (InCor), Clinics Hospital University of Sao Paulo Medical School (HCFMUSP), Av. Dr. Enéas Carvalho de Aguiar, 44 - Cerqueira César, São Paulo, SP 05403-900, Brazil; University of Sao Paulo Medical School (FMUSP), Av. Dr. Arnaldo, 455 - Cerqueira César, Pacaembu, SP 01246-903, Brazil; Polytechnique School, University of Sao Paulo (POLI USP), Av. Prof. Luciano Gualberto, 380 - Butantã, São Paulo, SP 05508-010, Brazil

**Keywords:** Chest X-ray, Aortic elongation, Deep learning, Explainable AI

## Abstract

**Aims:**

Aortic elongation can result from age-related changes, congenital factors, aneurysms, or conditions affecting blood vessel elasticity. It is associated with cardiovascular diseases and severe complications like aortic aneurysms and dissection. We assess qualitatively and quantitatively explainable methods to understand the decisions of a deep learning model for detecting aortic elongation using chest X-ray (CXR) images.

**Methods and results:**

In this study, we evaluated the performance of deep learning models (DenseNet and EfficientNet) for detecting aortic elongation using transfer learning and fine-tuning techniques with CXR images as input. EfficientNet achieved higher accuracy (86.7% ± 2.1), precision (82.7% ± 2.7), specificity (89.4% ± 1.7), F1 score (82.5% ± 2.9), and area under the receiver operating characteristic (92.7% ± 0.6) but lower sensitivity (82.3% ± 3.2) compared with DenseNet. To gain insights into the decision-making process of these models, we employed gradient-weighted class activation mapping and local interpretable model-agnostic explanations explainability methods, which enabled us to identify the expected location of aortic elongation in CXR images. Additionally, we used the pixel-flipping method to quantitatively assess the model interpretations, providing valuable insights into model behaviour.

**Conclusion:**

Our study presents a comprehensive strategy for analysing CXR images by integrating aortic elongation detection models with explainable artificial intelligence techniques. By enhancing the interpretability and understanding of the models’ decisions, this approach holds promise for aiding clinicians in timely and accurate diagnosis, potentially improving patient outcomes in clinical practice.

## Graphical Abstract

**Graphical Abstract:**
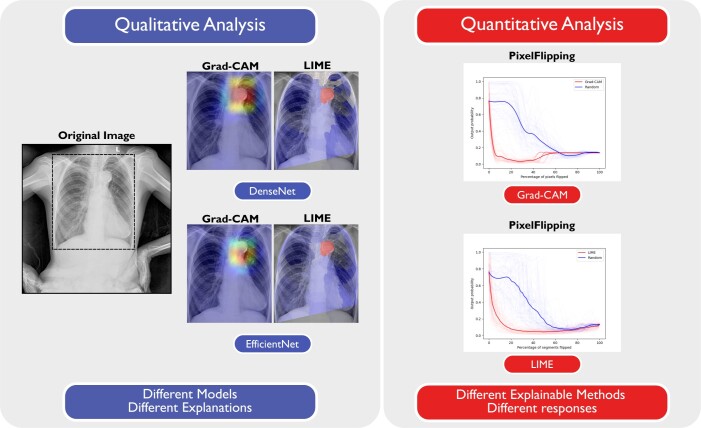

## Introduction

Aortic elongation is a medical condition where the aorta, the largest artery in the human body, is longer than normal.^1^ This can occur due to age-associated changes,^2^ congenital factors,^3–7^ or medical conditions that affect blood vessel elasticity, leading to cardiovascular diseases. Common diseases associated with aortic elongation include aortic aneurysms,^8^ Marfan syndrome,^6^ Ehlers–Danlos syndrome,^3^ Loeys–Dietz syndrome,^7^ and Turner syndrome.^4^ The severity and implications of aortic elongation can vary; while some cases may be asymptomatic, others can result in severe complications such as aortic aneurysms, dissection, rupture, and other cardiovascular conditions.^1^

Detection of aortic elongation can be achieved through various imaging examinations, including chest X-rays (CXR), computerized tomography, and magnetic resonance imaging. Advancements in the field of deep learning (DL) have significantly enhanced efforts to automate disease detection using these imaging modalities. Deep learning provides powerful tools for processing and analysing medical images, benefiting research focused on the automatic detection of diseases.

Convolutional neural networks (CNNs),^9,10^ a type of DL model, are commonly used in computer vision tasks such as image and video recognition. Convolutional neural networks can extract features from raw data without prior knowledge of the input data, making them particularly useful for image recognition tasks where traditional feature extraction methods may not be effective. They learn multiple layers of abstraction that represent features at increasing levels of complexity, enabling the recognition of complex patterns and objects. Moreover, CNNs can identify patterns that may be difficult for the human eye to notice by learning from large amounts of data. Overall, DL has the potential to greatly improve the accuracy and efficiency of medical imaging, leading to better patient outcomes and more effective healthcare.

However, it is paramount to carefully validate and interpret the results of DL models, as they can be sensitive to biases in the training data and may not always generalize to new patient populations.^11^ Furthermore, the black-box nature of DL models hinders transparency in their decision-making processes, despite their high classification accuracy.^12^ This lack of interpretability limits the application of artificial intelligence (AI) systems in hospitals.^12^ Recently, this issue has garnered significant attention, raising questions about the use of AI systems in high-risk areas such as medicine.^11,13^ Consequently, many researchers are developing techniques to understand how DL models reach their decisions, known as explainable artificial intelligence (XAI).^14–17^ Some of these techniques rely on gradient analysis,^16,17^ which can be difficult to interpret, while others are based on perturbation,^14,15^ which can be specific to particular instances.

Despite these interpretability challenges, integrating DL models with medical knowledge presents promising opportunities, particularly in radiology. Since CXR exams are quick and inexpensive, their use for detecting aortic elongation is important as this condition can indicate a range of diseases. Low-income hospitals that may not have access to experienced radiologists could benefit from implementing an automatic detection model for this condition, reducing the burden on their medical infrastructure. Additionally, even experienced experts are susceptible to errors; thus, a DL model can assist in the arduous and time-consuming task of interpreting and evaluating CXR images. By quickening the diagnostic process and lessening the workload of doctors, integrating DL models with medical knowledge can enhance medical staff performance and reduce patient waiting times.

In this study, we implemented two DL models to detect aortic elongation using CXR images from publicly available and private datasets, employing transfer learning and fine-tuning approaches. To explain our models’ predictions, we used gradient-weighted class activation mapping (Grad-CAM) and local interpretable model-agnostic explanations (LIME), two widely known XAI methods, to visualize which parts of the CXR are most important when predicting aortic elongation. Additionally, we used the pixel-flipping metric to quantitatively evaluate the explainable methods, providing qualitative and quantitative insights into the models’ predictions. Our study offers the following key contributions:

- Development of DL models utilizing transfer learning and fine-tuning techniques to accurately identify aortic elongation in CXR images
- Implementation of Grad-CAM and LIME explainability methods to gain insights into the decision-making processes of the DL models
- Comparative analysis of two distinct DL models based on their performance metrics and explainability outputs
- Utilization of the pixel-flipping quantitative metric to evaluate the interpretability and reliability of the explanations provided by XAI methods

## Methods

**Figure 1:**
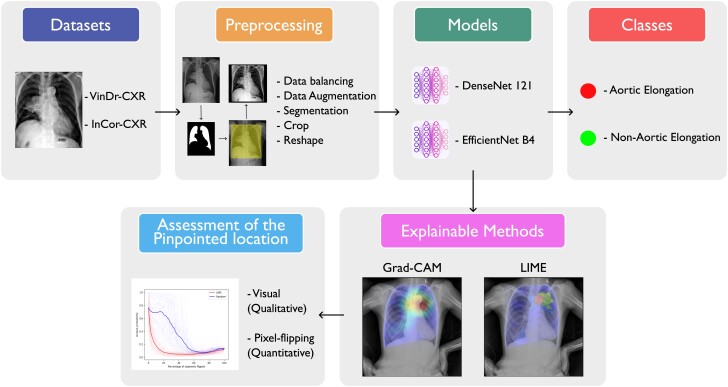
General structure of the proposed methodology. Grad-CAM, gradient-weighted class activation mapping; LIME, local interpretable model-agnostic explanations.

The overall structure of our proposed method is illustrated in *Figure 1*. We utilized two CNNs, namely, DenseNet and EfficientNet. The training process began with initializing the models using ImageNet weights. We then employed transfer learning using a publicly available CXR data set to enable our models to learn the new domain. Following this, we performed fine-tuning with a private CXR data set. To evaluate the performance of our models, we used a five-fold cross-validation strategy. After assessing their performance, we retrained the models on the entire data set, bypassing the cross-validation process. This allowed us to develop comprehensive models, which were subsequently used for the proposed XAI methods. Finally, we conducted a visual analysis of the interpretations provided by the explainable methods Grad-CAM and LIME, alongside a quantitative analysis using the pixel-flipping method, on a subset of the test set from our private data set.

### Datasets

The VinDr-CXR dataset^18,19^ comprises CXR scans retrospectively obtained from two major hospitals in Vietnam. All images are in Digital Imaging and Communications in Medicine (DICOM) format and in posteroanterior (PA) view. This dataset contains 18 000 images manually annotated by a group of radiologists. It is divided into a training set with 15 000 scans, each independently labelled by 3 radiologists, and a test set with 3000 scans labelled by the consensus of 5 radiologists. Out of the 15 000 exams, 10 606 were labelled as ‘normal’, while 4376 were identified with ‘abnormal conditions’, with 2350 labelled as having aortic elongation and 2026 showing other abnormalities.

To address the class imbalance issue, we randomly selected 4376 exams from the ‘normal’ class to match the number of abnormal cases, resulting in a final dataset of 8752 exams. This ensured a balanced representation of both normal and abnormal cases. Note that due to the lack of patient’s ID information, images from the same patient might appear in both train and test sets.

In addition to the VinDr-CXR dataset, we used a private CXR dataset (InCor-CXR) that contains retrospective data collected from the picture archiving and communication system of a tertiary referral hospital in Brazil specialized in cardiology (Heart Institute Hospital). The data were collected from 2016 to 2019 and included patients older than 18 years. All CXR images are in DICOM format and in a PA view. The diagnostic reports presented in a structured text format were created by experienced radiologists during routine clinical practice and independently confirmed by two specialists to ensure accuracy. This private dataset contains 473 exams labelled as aortic elongation. To balance the non-aortic elongation class, we included 473 exams, equally sampled from those labelled as normal and abnormal conditions. The InCor-CXR dataset complies with all relevant ethical regulations and was approved by the Institutional Review Board (IRB) under registration 45070821.3.0000.0068. *Table 1* summarizes the information regarding both datasets.

**Table 1:** Number of chest X-ray exams labelled as aortic elongation from VinDr-CXR dataset and our InCor-CRX private database

	VinDr-CXR	InCor-CXR
Aortic elongation	2350	473
Non-aortic elongation	6402	473
Total	8752	946

### Image pre-processing

**Figure 2:**
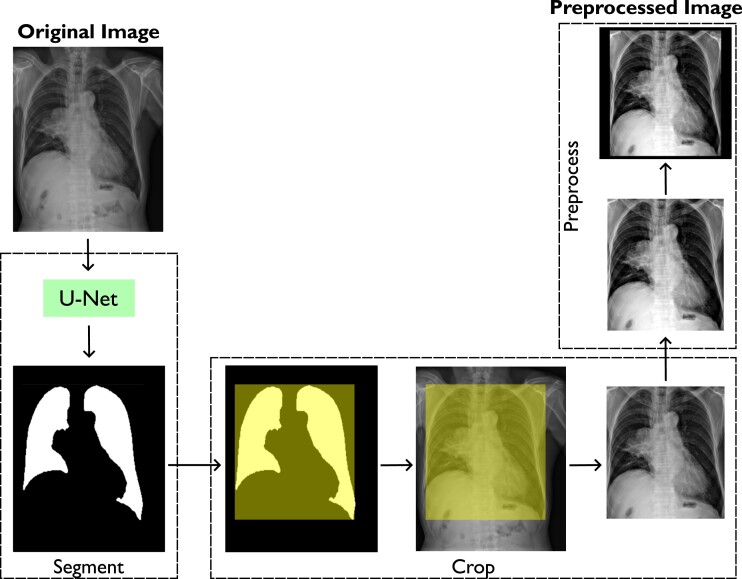
Steps for the proposed pre-processing task: (i) chest cavity segmentation, (ii) cropping, (iii) contrast stretching, (iv) fill borders to get squared images, and (v) resize.

Our pre-processing steps are based on previous works^20,21^ focused on cardiomegaly classification. To enhance training efficiency and reduce computational costs, we utilized a previously developed and validated model^22^ that segments the lungs using a fine-tuned UNet-based CNN. This model creates a binary mask of the chest cavity region. We used the extreme points of the lung mask to create a bounding box, which allowed us to crop the chest cavity, segmenting only this region of interest that was used as input in our classification model. We rescaled the intensity of the cropped image with a contrast stretching method, including all intensities within the 1st and 99th percentiles of the image histogram. The image was then converted into a square format through zero padding, to ensure the preservation of tissue morphology, as networks generally require square input images. *Figure 2* displays the described pre-processing steps.

### Deep learning model

**Figure 3:**
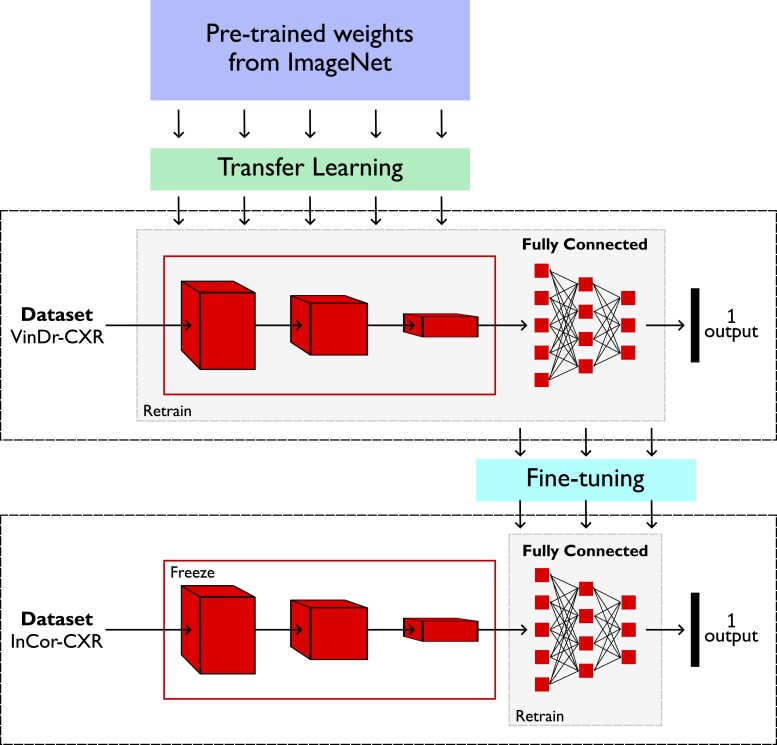
The transfer learning and fine-tuning techniques used in the development of the proposed convolutional neural network models.

The small size of datasets in DL for analysing medical images is a significant constraint. Consequently, training a CNN from scratch can be challenging.^23^ Transfer learning is a common solution. To do so, we use a pre-trained network as a feature extractor and retrained it with the data from the new domain, such as CXR images. We initiated the training with the ImageNet weights.^24^ Then, we trained the models using the VinDr-CXR dataset to learn the patterns specific to the CXR domain. Subsequently, we performed fine-tuning using our private InCor-CXR dataset. Only the fully connected layers of the model were unfrozen during this fine-tuning process. *Figure 3* illustrates the overall proposed model based on transfer learning and fine-tuning techniques.

For our experiments, we selected two DNN architectures widely used for CXR classification: DenseNet 121 and EfficientNet B4 architectures. We used the default Keras architecture topology with an input resolution of 384 × 384 × 3 for DenseNet 121 and an input resolution of 380 × 380 × 3 for EfficientNet B4. For the fully connected layers, we employed a customized two-layer perceptron with dropout regularization of 30%, Rectified Linear activation in the first layer (*n* = 256), and a sigmoid function in the final layer. Our model was trained over 40 epochs, with a 16 batch size per step using Adam optimizer with a learning rate of 0.0001 and a callback to reduce it by a factor of 0.2 every 6 epochs in case of no improvement in validation loss. Moreover, the data were augmented via (i) rotation, (ii) horizontal flipping, and (iii) vertical flipping. Our data augmentation strategy involved applying a 2× augmentation factor to the major class and an appropriately computed factor to the minor class. The purpose of this approach was to balance class representation and address any potential class imbalance issues.

We implemented a robust five-fold cross-validation strategy during the fine-tuning step. To evaluate the models, we assessed five metrics: accuracy (Acc), precision (Prec), sensitivity (Se), specificity (Spe), F1 score (F1), and area under the receiver operating characteristic (AUROC).

We utilized a cross-validation approach to estimate the performance of our models, which involved splitting our data into multiple subsets to train and evaluate the models iteratively. Once we obtained an understanding of their performance, we proceeded to retrain the models using the entire dataset, without the cross-validation process. This allowed us to create complete models, which were subsequently applied to perform the proposed XAI methods.

Our experiments were conducted using a Foxconn High-Performance Computer (HPC) M100-NHI with an 8 GPU cluster of 16 GB NVIDIA Tesla V100 cards. The methodology was implemented using the Python framework and Keras v2.4.3 with TensorFlow backend v2.3.0.

### Explainable artificial intelligence methods

We employed two commonly used local explainable methods to analyse individual CXR images: LIME^15^ and Grad-CAM.^16^ These methods provide valuable insights into the decision-making mechanisms of complex machine learning models. The outcome of these analyses is visualized in a map where the most important regions are highlighted in red for both Grad-CAM and LIME methods.

LIME^15^ is a model-agnostic method that explains the predictions of a machine learning model in an easily interpretable manner. It works by creating a local linear model around a specific data point to approximate how the original model makes its predictions. It then identifies the features, or superpixels (segments on the image instance) in the context of image inputs, of the input that are most important in determining the prediction outcome. In our work, we used linear regression as the surrogate model and segmented the images with a *quickshift* segmentation method from the *skimage* segmentation library in Python, with the following parameters: kernel_size = 5, max_dist = 100, and ratio = 0.5. We used 1000 perturbations to perform LIME.

Gradient-weighted class activation mapping^16^ is a visualization method that helps understand the regions of an image that contribute to a CNN’s prediction. It uses the gradients of the predicted class with respect to the feature maps of the last convolutional layer to generate a heat map, highlighting the regions of the input image most relevant to the final prediction.

Both LIME and Grad-CAM are important tools for XAI, as they help identify the features or regions of the input that the model relies on to make predictions. These methods can be used to detect potential errors in the model, identify biases, or simply provide insights into how the model processes input data. By offering a more transparent and interpretable view into the inner workings of machine learning models, LIME and Grad-CAM contribute to building more trustworthy and reliable AI systems.

### Quantification of explainable artificial intelligence methods

The pixel-flipping method^25,26^ is a technique used to quantify the explainability of AI methods that operate on image data, particularly in the context of computer vision tasks such as image classification. The goal of this method is to assess how sensitive the AI model’s predictions are to changes in individual pixels of an input image, providing insights into the model’s decision-making process and which image regions are important for its predictions. The pixel-flipping method typically involves the following steps:

- **Input image selection:** a set of input images from the InCor-CXR dataset used to test the AI model are selected for analysis. These images should represent the task of interest and cover a range of different samples to ensure a comprehensive assessment. Here, we selected a subset of the InCor-CXR test set with 70 CXR exams labelled as aortic elongation.
- **Perturbation:** each selected image is then perturbed by flipping the value of individual pixels, either by inverting their intensity (e.g. changing a pixel from black to white or vice versa) or by changing their colour value (e.g. changing a pixel from red to green or vice versa). In this work, we set a pixel to zero for the Grad-CAM method or set a segment to zero for the LIME method. This perturbation is done based on their importance as determined by the explainable methods, ordered from the most relevant to the least according to the particular explanation, showing how quickly the prediction score decreases. Likewise, for a baseline, we randomly selected the pixels or segments for comparison.
- **Prediction evaluation:** the perturbed images are then fed into the DL model, and the resulting predictions are compared with the original predictions on the unperturbed image. The discrepancy between the original and perturbed predictions is used as a measure of the model’s sensitivity to pixel-level changes. For example, if flipping a certain pixel significantly changes the model’s prediction, it indicates that the model may rely heavily on that pixel for its decision-making process. In this work, we present the curves of the DL model output score for the perturbed images.
- **Interpretation:** finally, the results of the pixel-flipping analysis can be interpreted to gain insights into the model’s behaviour. For example, if certain pixels have a high impact on the model’s predictions, it may indicate that those pixels are important for the model’s decision-making process and should be further investigated. This information can help understand the inner workings of the DL model and identify potential biases or limitations in its decision-making process.

Overall, the pixel-flipping method is a useful tool for quantifying the explainability of AI methods in computer vision tasks by assessing their sensitivity to pixel-level changes. In our implementation, for LIME, we gradually removed (set a segment to zero) the segments of an individual input image, ordered from most relevant to least according to the particular explanation, showing how quickly the prediction score decreases. For Grad-CAM, we removed 10, 20, and up to 100% of the pixels, based on the most relevant on the resulting heat map. Pixel-flipping curves are computed for a subset of the test set of individual images and then averaged to create a mean curve. Finally, for comparison (baseline), we performed pixel-flipping by randomly removing the segments and pixels for both LIME and Grad-CAM methods. By randomly removing segments, we are essentially creating a scenario where segments are altered without regard to their relevance to the model’s decision. This random alteration serves as a comparison to highlight that not all segments are equally important.

## Results

**Figure 4:**
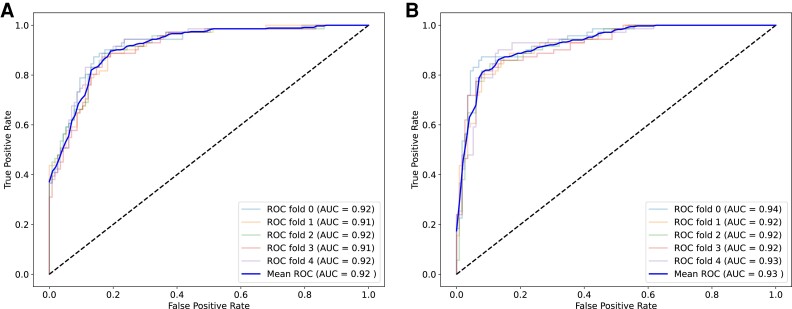
The receiver operating characteristic curves of (*A*) DenseNet and (*B*) EfficientNet. AUC, area under the curve; ROC, receiver operating curve.

The performance metrics of our models are outlined in *Table 2*, and their corresponding receiver operating characteristic curves are displayed in *Figure 4*. It can be observed that the EfficientNet model achieved better results than the DenseNet model in identifying aortic elongation. These results demonstrate the effectiveness of our approach in developing a reliable model for aortic elongation detection.

**Table 2:** Performance of our proposed models for the identification of aortic elongation on chest X-ray images

Model	Acc	Prec	Se	Spe	F1	AUROC
EfficientNet	0.867 (0.021)	0.827 (0.027)	0.823 (0.032)	0.894 (0.017)	0.825 (0.029)	0.927 (0.006)
DenseNet	0.833 (0.010)	0.734 (0.016)	0.885 (0.023)	0.802 (0.019)	0.802 (0.011)	0.915 (0.006)

Results are presented as mean (SD).

**Figure 5:**
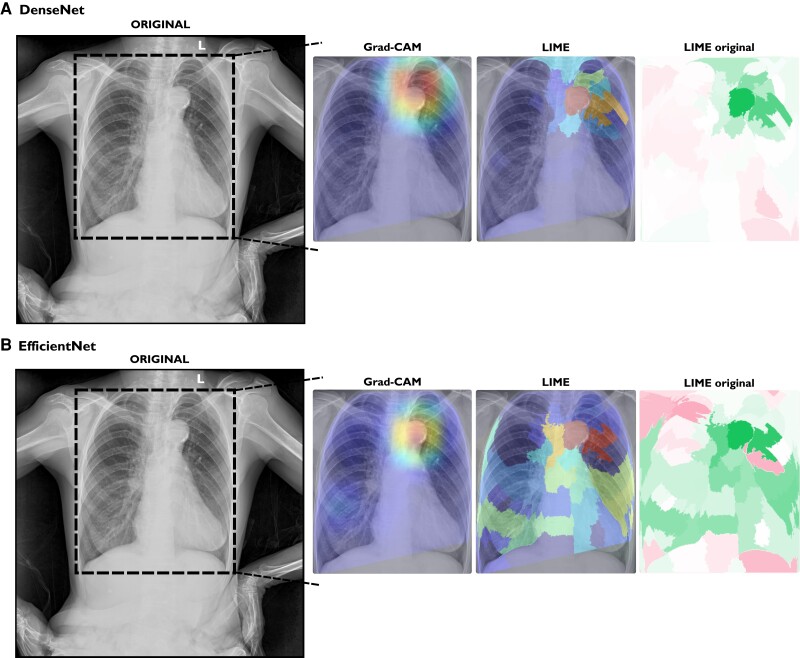
Explainable methods’ results for an exam correctly labelled as aortic elongation for both models. (*A*) DenseNet prediction probability of aortic elongation: 99.44%. (*B*) EfficientNet prediction probability of aortic elongation: 99.64%. In the original image, a region of interest is demarcated by a dashed box. The output of each method, gradient-weighted class activation mapping and local interpretable model-agnostic explanations, is superimposed on the chest X-ray image. The most relevant regions are highlighted in red, while the least relevant regions are indicated in blue. The ‘LIME original Mask’ refers to the mask generated by the local interpretable model-agnostic explanations method. Grad-CAM, gradient-weighted class activation mapping; LIME, local interpretable model-agnostic explanations.

*Figure 5* displays the results of the Grad-CAM and LIME methods for an exam labelled as aortic elongation (DenseNet prediction probability 99.6%, EfficientNet prediction probability 97.8%). Both explainable methods correctly identified the location of the aortic elongation.

**Figure 6:**
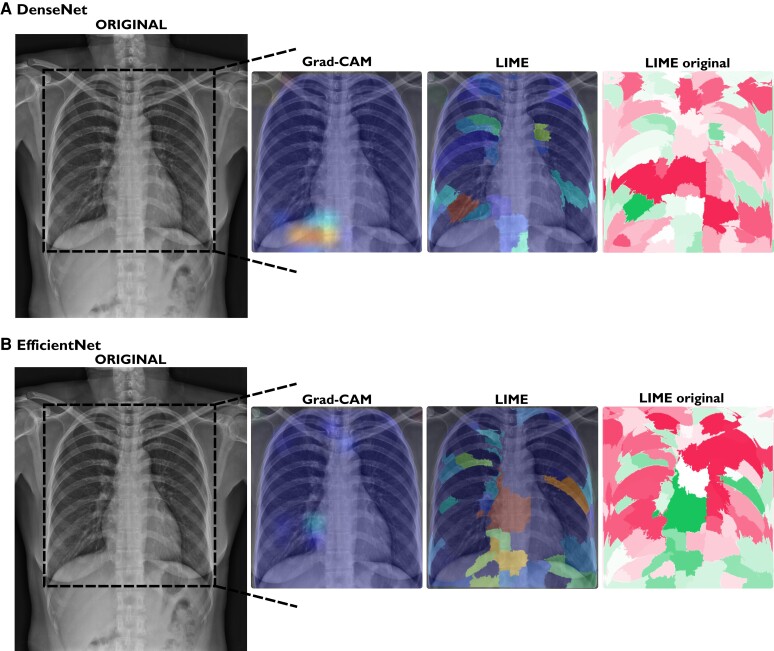
Explainable methods’ results for an exam correctly labelled as non-aortic elongation. (*A*) DenseNet prediction probability of aortic elongation: 0.60%. (*B*) EfficientNet prediction probability of aortic elongation: 3.06%. Grad-CAM, gradient-weighted class activation mapping; LIME, local interpretable model-agnostic explanations.

Similarly, *Figure 6* shows the results of the explainable methods for an exam labelled as non-aortic elongation (DenseNet prediction probability, 0.3%; EfficientNet prediction probability, 0.8%). Neither method highlighted any specific region since this is a normal CXR exam.

**Figure 7:**
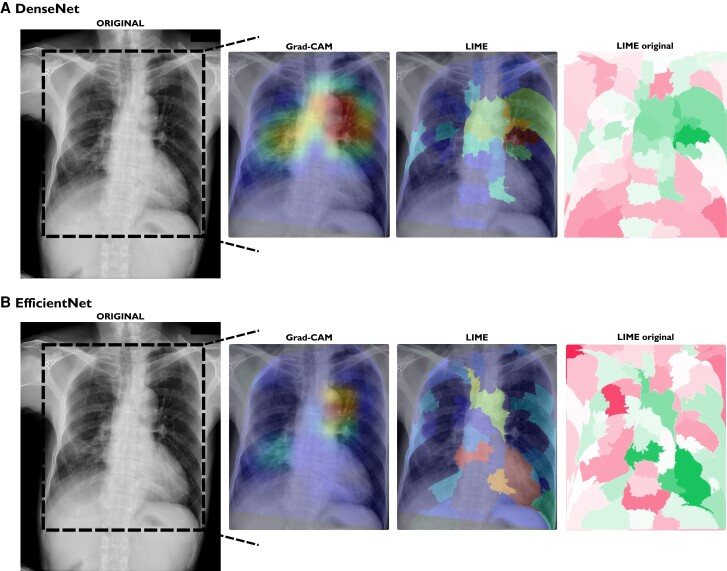
Explainable methods’ results for an exam wrongly labelled as aortic elongation. (*A*) DenseNet prediction probability of aortic elongation: 80.16%. (*B*) EfficientNet prediction probability of aortic elongation: 92.81%. Grad-CAM, gradient-weighted class activation mapping; LIME, local interpretable model-agnostic explanations.

*Figure 7* presents the results of an exam that was wrongly predicted as aortic elongation but is labelled as cardiomegaly in the dataset (DenseNet prediction probability, 59.4%; EfficientNet prediction probability, 79.2%). Here, all explainable methods identified the aorta region, except for the LIME method on the EfficientNet model, indicating the heart as the most important segment.

**Figure 8:**
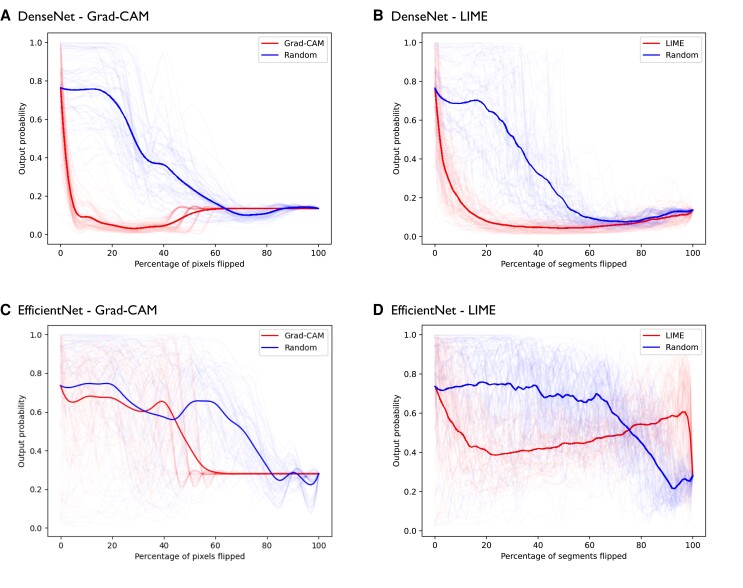
Pixel-flipping curves for a subset of individual chest X-ray exams (thin lines) and their average mean curves (thick line) for both explainable methods, gradually removing the segments ordered from the most relevant (red lines) or randomly removing the segments (blue lines). (*A* and *C*) Grad-CAM, gradient-weighted class activation mapping; (*B* and *D*) LIME, local interpretable model-agnostic explanations.

To assess the reliability of our studied explainable methods, we utilized the pixel-flipping method. The results of this quantitative method are illustrated in *Figure 8*. There are significant differences in the results of both models. When relevant pixels/segments are removed, the DenseNet model experiences a rapid decline in its prediction performance (output score) for both Grad-CAM and LIME methods, which aligns with the desired behaviour. However, for the EfficientNet model, the pixel-flipping method didn’t present the same distinctive difference from the baseline of random removal.

## Discussion

We have developed a comprehensive strategy integrating the aortic elongation detection model with XAI methods to analyse CXR images. This approach has the potential to enhance diagnostic accuracy and aid in the early detection of this cardiac condition. Through transfer learning and fine-tuning processes, our models accurately identified aortic elongation. By using explainable methods, we can provide valuable insights for physicians into the decision-making process of our models. Given that CXRs are cost-effective and time-efficient, they serve as an ideal initial screening tool. Utilizing this approach, high-risk patients can be efficiently triaged during routine care, enabling timely lifestyle modification counselling and further examinations or interventions if necessary.

In a qualitative analysis, Grad-CAM and LIME explainable methods correctly pinpointed the expected aorta region on exams correctly predicted as aortic elongation. In the first example (*Figure 5*), it is interesting to observe that both models presented different explanations. One could argue that different models learn the problem (aortic elongation prediction, here) differently, and the visual explanations could assess if they learned the problem correctly. In the second example (*Figure 6*), we can see that for this normal CXR, the models seem to not know where to look on the image. One could suggest that the model didn’t find the aorta problem, making the explainable methods highlight spurious regions of the image. Moreover, again, we can see the difference between both models, where DenseNet (LIME) only highlights a few segments, whereas EfficientNet (LIME) indicates more regions on the CXR. In the case where the models wrongly predicted aortic elongation (*Figure 7*), both models indicated the aorta region as the most relevant.

Quantitatively, it is expected that the explainable methods that accurately highlight the image pixels/segments influencing the model’s decision will exhibit a rapid decline in prediction performance (mean output score) as these pixels/segments are progressively perturbed (set to zero). To establish a baseline, random explanations (blue lines) are also included in *Figure 8*, as it is anticipated that perturbing image pixels/segments randomly will result in a slower decrease in performance. In addition, it is important to clarify that the results obtained through the pixel-flipping method reflect the output score of the models and not their accuracy. The output score represents the confidence or certainty assigned by the model to a specific prediction. It is distinct from accuracy, which measures the overall correctness of the model’s predictions.

Based on our findings in *Figure 8*, several observations can be highlighted:

- The DenseNet model demonstrated the expected behaviour for both explainable methods.
- Both Grad-CAM and LIME on the DenseNet model significantly distinguished itself from the baseline (random removal). Removing segments rapidly led to a drop in prediction performance and the removal of the most important segments made the model change prediction to non-aortic elongation. With the removal of the final segments, the performance reached a basal level for both explainable methods.
- The Grad-CAM method on the EfficientNet model did not clearly differentiate itself from the baseline (random removal); however, when 50% of the segments are removed, the model changes prediction to non-aortic elongation.
- For the LIME on the EfficientNet, the output score drops at first but then starts to rise above 0.5 until it drops at the very end. This suggests that if we take a CXR image and set, e.g. 90% pixels/segments to zero, the model, on average, would classify it as aortic elongation using a threshold of 0.5.

One possible interpretation of these pixel-flipping curves could be that a binary model that consistently outputs near-zero scores when all segments are set to zero implies that the model has learned to distinguish between the absence of relevant features and the presence of those indicative of the target class. In other words, if the model consistently outputs a high score (close to 1) when the features related to the target class are present and a low score (close to 0) when the features are absent, it indicates that the model has learned to associate the presence of those specific features with the target class. This behaviour would suggest that the model has effectively learned to discriminate between aortic elongation and other classes or background noise. It could demonstrate a robust understanding of the distinguishing features of the target class and could reflect a good capability in solving the classification problem. However, it’s important to note that a completely black image (with all pixels set to zero) falls outside the domain on which the model was trained. Therefore, the model is not fitted to recognize this pattern since it hasn’t learned it. These observations provide valuable insights into the behaviour and performance of the DenseNet and EfficientNet models when subjected to the explainable and pixel-flipping methods.

To the best of our knowledge, there are only a limited number of studies that have focused on the automated detection of aortic elongation specifically from CXR images. Some studies employ a multi-label paradigm, where aortic elongation may be included as one of the labels depending on the dataset. However, the number of works specifically targeting the detection of aortic elongation in CXR remains relatively scarce. Rosenwasser *et al.* (2022)^27^ used an Inception V3 and a DenseNet169 model on the VinDr-CXR dataset, relating this cardiac condition with Marfan syndrome. Similarly, Lee *et al.* (2022)^28^ propose the detection of aortic dissection based on a ResNet 18 CNN, using Grad-CAM to visually interpret their results. Hence, it is important to emphasize the significance of automating the identification of these conditions, given their potential implications for patient health and the effectiveness of medical interventions.

Many remote hospitals lack access to radiologists, making computer-aided diagnostic systems crucial for accurate results. These systems aid inexperienced doctors, reduce expenses, prioritize critical cases, and shorten wait times. However, their adoption in clinical practice is rare due to inconsistencies in existing datasets extracted from radiology reports using natural language processing methods, scarcity of labelled CXR datasets, research focusing more on model performance than practical application, and the complexity of machine learning algorithms hindering interpretability.

It is indispensable for models to generate explanations about their decision-making. The issue of interpretability cannot be a limiting factor for systems already developed. We must make use of the great existing capacity of AI systems to generate more efficient and reliable models. Gradient-weighted class activation mapping and LIME methods can provide valuable insights into how AI models make decisions and identify important image regions for their predictions, which can be useful for improving model transparency, fairness, and trustworthiness. Moreover, pixel-flipping can give a quantitative metric to the explainable methods, allowing assessing their interpretations.

In summary, our results demonstrate the effectiveness of our approach in developing a reliable model for aortic elongation detection. The high accuracy, precision, and AUROC scores achieved by the DenseNet model, along with supporting visualizations from the explainable methods, underscore its potential value in clinical practice. Further research and development in this area could lead to improved diagnostic capabilities and enhanced patient care in the field of cardiac imaging.

## Conclusions

Our study presents a strategy for analysing CXR images to detect aortic elongation using DL models and XAI methods. Our approach demonstrates promising results in enhancing diagnostic accuracy and aiding early detection. By leveraging transfer learning techniques, our models accurately identify aortic elongation, and explainable methods provide valuable insights into their decision-making processes for physicians. Our findings highlight the potential of AI-driven diagnostic systems to streamline healthcare processes and prioritize patient care. Continued research in this area is crucial for further advancements in cardiac imaging and the broader adoption of AI technologies in clinical practice.

## Data Availability

The VinDr-CXR dataset underlying this article is available in VinBigData repository, at https://doi.org/10.48550/arXiv.2012.15029. The InCor-CXR dataset cannot be shared publicly due to internal hospital policies. The data will be shared on reasonable request to the corresponding author.
